# A Proteomic Approach to Identify Zein Proteins upon Eco-Friendly Ultrasound-Based Extraction

**DOI:** 10.3390/biom11121838

**Published:** 2021-12-06

**Authors:** Laura Darie-Ion, Madhuri Jayathirtha, Gabriela Elena Hitruc, Marius-Mihai Zaharia, Robert Vasile Gradinaru, Costel C. Darie, Aurel Pui, Brindusa Alina Petre

**Affiliations:** 1 Faculty of Chemistry, Al. I. Cuza University of Iasi, 11, Carol I Boulevard, 700506 Iasi, Romania; laura.ion@uaic.ro (L.D.-I.); robert.gradinaru@uaic.ro (R.V.G.); aurel@uaic.ro (A.P.); 2 Department of Chemistry & Biomolecular Science, Clarkson University, 8 Clarkson Avenue, Potsdam, NY 13699, USA; jayathm@clarkson.edu (M.J.); cdarie@clarkson.edu (C.C.D.); 3 Petru Poni Institute of Macromolecular Chemistry, 41A Gr. Ghica Voda Alley, 700487 Iasi, Romania; gabihit@icmpp.ro (G.E.H.); zaharia.marius@icmpp.ro (M.-M.Z.); 4 Center for Fundamental Research and Experimental Development in Translation Medicine–TRANSCEND, Regional Institute of Oncology, 700483 Iasi, Romania

**Keywords:** zein, ultrasound extraction, electrophoresis, mass spectrometry, atomic force microscopy

## Abstract

Zein is a type of prolamin storage protein that has a variety of biomedical and industrial applications. Due to the considerable genetic variability and polyploidity of the starting material, as well as the extraction methods used, the characterization of the protein composition of zein requires a combination of different analytical processes. Therefore, we combined modern analytical methods such as mass spectrometry (MS), Sodium dodecyl sulfate polyacrylamide gel electrophoresis (SDS-PAGE), atomic force microscopy (AFM), or Fourier transform infrared spectroscopy–attenuated total reflectance (FTIR-ATR) for a better characterization of the extracted zein. In this study, we present an enhanced eco-friendly extraction method, including grinding and sieving corn seeds, for prolamins proteins using an ultrasonic extraction methodology. The use of an ultrasonic homogenizer, 65% ethanol extraction buffer, and 710 µm maize granulation yielded the highest protein extraction from all experimental conditions we employed. An SDS PAGE analysis of the extracted zein protein mainly revealed two intense bands of approximatively 20 and 23 kDa, suggesting that the extracted zein was mostly α-zein monomer. Additionally, MS analysis revealed as a main component the α-zein PMS2 (Uniprot accession no. P24450) type protein in the maize flour extract. Moreover, AFM studies show that extracting zein with a 65% ethanol and a 710 µm granulation yields a homogeneous content that could allow these proteins to be employed in future medical applications. This research leads to a better understanding of zeins content critical for developing new applications of zein in food and pharmaceutical industries, such as biocompatible medical vehicles based on polyplexes complex nanoparticles of zein with antimicrobial or drug delivery properties.

## 1. Introduction

Zein proteins, which belong to the prolamine protein class and were first identified in 1821 by Gorham [1], account for more than 60% of the total endosperm protein in cereals. This type of storage protein, with a high leucine (19.3%), proline (9%), and glutamine (21.4%) content [2,3], low essential amino acids content such as tryptophan (0%) and lysine (0.4%) [4], are soluble only in organic solvent aqueous solutions [5]. As a result, zein demonstrates unique amphiphilicity, the molecular basis for self-assembly molecular structures. Zein proteins also adopt a secondary structure that is high in α-helix (30–80%) and may be folded into a unique tertiary structure with a regular geometry [6] that helps further self-assembling layers. Moreover, based on their solubility and amino acid sequence, the zein was classified in different isoforms such as α-zein (80%), β-zein (10–15%), γ-zein (10–15%), and δ-zein types [7,8].

Zein proteins have found wide applications as excipients in the production of inks [9], adhesives [10], water resistant paper coating [11,12], plastic-free replacement [13], encapsulated foods [14], and a controlled release matrix of active medical components [15,16,17,18]. Moreover, among various protein sources, zein (the primary storage protein in corn endosperm) has been extensively studied for its capacity to create innovative polymeric films, a feature we want to explore in the future [19,20,21]. Researchers’ interest in plant proteins has increased due to their diversity and applicability, and as such, several studies show that whey protein forms nanofibers as carriers of lipophilic bioactive substances [22,23], soy lipophilic protein forms nanoparticles as a conjugated linoleic acid vehicle [24] and nanoparticles based on linseed protein [25]. Above all plant-proteins, we use zein in our research as an ideal candidate due to its broad characteristics, including excellent plasticity, harmlessness, and biologically biodegradability [26]. Moreover, conlinin (16–18 kDa), a water-soluble protein in flaxseed and the correspondent of zein proteins in corn, has the disadvantage of not being resistant to the enzymes of the digestive tract by the presence of 16 residues of Lys/Arg if compared with zein proteins (only 4 basic residues) [25]. These features make zein a major biocomponent for controlled drugs delivery devices and urges researchers to provide affordable extraction methods to facilitate the cost-effective production of large-scale zein production.

Dry-milled corn (DMC), corn gluten meal (CGM), and corn fermentation coproduct (DDGS) are the three maize components from which zein was previously extracted using hazardous chemicals [27]. Following the discovery of zein, numerous researchers became interested in expanding the extraction procedures in order to obtain more homogenous zein content [28,29,30]. However, the previously used methods have diverse drawbacks, including the use of toxic solvents (e.g., toluene, ether, and hexane), high temperatures, extensive extraction times, and additional purifications steps following organic extraction [31,32,33]. Therefore, improvements in zein extraction (e.g., solvent combinations, reducing agents, or ultrasonic techniques) still need to be evaluated. The ultrasonic technique proved to be a quick, non-destructive, and low-cost approach for extracting proteins from diverse biological samples. It was shown that an ultrasound-assisted approach for extracting defatted wheat germ proteins increased their content from 37% to 57% [34].

Furthermore, due to the extensive genetic variety and polyploidity of the starting material, as well as the extraction methods used, we previously revealed that zein proteins are a group of comparable polypeptides still showing non-homogenous fractions [35]. Therefore, analyzing protein composition involves the employment of multiple sophisticated analytical processes due to the complexity of zein and the extraction protocol (solvents, concentration, grinding, and degreasing). Due to their high sensitivity and mass accuracy, MS is complementarily used for the exact identification of separated proteins by SDS-PAGE. An ionization technique such as MALDI-ToF MS (matrix-assisted laser desorption ionization mass spectrometry) was used to better identify and characterize prolamin proteins in previous studies [7,36]. Although it is well known that a protein structure impacts its function and stability, it is critical to thoroughly explore the self-assembling behaviors of zein molecules. AFM is used to assess the surface morphology and three-dimensional structure of food products at a nanoscale range without destroying their content, allowing the single molecule investigation of a food complex sample. In previous studies, the morphologies of commercial zein at different concentrations (0.01–1.00% or <0.01%, *w/v*) in an aqueous ethanol solution of 70% or 75% (*v/v*) were investigated using tapping mode AFM [37,38,39].

For a better characterization of the extracted zein, we applied advanced analytical approaches such as ESI (electrospray ionization) and MALDI-ToF mass spectrometry, sodium dodecyl sulfate polyacrylamide gel electrophoresis, atomic force microscopy, and infrared spectroscopy fourier transform infrared spectroscopy–attenuated total reflectance.

Therefore, we report here a proteomic approach to identify zeins upon eco-friendly ultrasound-based extraction from a hybrid maize kernel variant KWS 3381. In detail, we describe an optimized extraction procedure that involves grinding and sieving corn seeds repeatedly to create maize flours protein compositions dependent on particle hardness, followed by the aqueous ethanol ultrasound-assisted extraction and investigation of physicochemical properties of the extracted zein.

## 2. Materials and Methods

### 2.1. Materials

Hybrid corn, KWS 3381, FAO group 450 was obtained from Monsanto, Bucharest, Romania. Commercial α-zein (Z3625-500G, batch no. SLBQ1626V) from maize, bovine serum albumin (BSA), ethanol, acetone, methanol, sodium dodecyl sulfate (SDS), glycerol, ammonium bicarbonate (NH_4_HCO_3_), iodoacetamide (IAA), trifluoroacetic acid (TFA) and the matrix, and 2-hydroxy-5-methoxybenzoic acid (DHB) were purchased from Sigma Aldrich (St. Louis, CA, USA). Protein assay dye reagent was obtained from Bio-Rad (Munich, Germany). Tris hydrochloride and dithiothreitol (DTT) were purchased from Merck (Darmstadt, Germany). The Coomassie Brilliant Blue R-250 Dye was purchased from Carl Roth (Karlsruhe, Germany). A PageRuler™ Unstained Protein Ladder from Thermo Scientific™ (Waltham, MA, USA) was used as protein marker. All reagents were of analytic purity and used without further purification. The solutions were prepared using deionized water (18.2 MΩ cm) from a Milli-Q system (Merck, Darmstadt, Germany).

### 2.2. Zein Protein Extraction

The milling, sieving, and defatting of the flour was performed as previously described [3,35]. Briefly, 150 g of untreated hybrid corn seeds were milled using a tripod portable flour mill (MB03, 1500 rpm, IPEE, Bucharest, Romania). Using a vibratory sieve shaker (AS 200 basic, Retsch, Haan, Germany) with two different meshes, 250 and 710 µm, the resulting flour was sieved for 7 min at 60 rpm. Furthermore, using a Soxhlet extractor (StemMart, Shirley, NY, USA) the flour fractions were defatted using petroleum ether. The defatted flour was then collected and dried in a laboratory oven (LDO-080F, Labtech, Sorisole, Italy) for 24 h at 100 °C. The extracted and defatted flour samples (300 mg) were suspended in 3 mL of 65% (*v/v*) (notated 65/250 and 65/710) and 95% (*v/v*) (notated 95/250 and 95/710) aqueous ethanol solution, respectively, and sonicated. A comparison of zein extraction methods was carried out employing an Ultrasonic homogenizer Cole-Parmer, (Vernon Hills, IL, USA) and an ultrasound bath, J.P. Selecta Ultrasons system, 40 kHz; Barcelona, Spain, for 15 min. The pellets were discarded after centrifugation for 15 min at 15,000 rpm (HettichMikro 22R centrifuge, Tuttlingen, Germany) at RT, and the supernatants were collected and stored at −4 °C for further analyses.

Calibration curves were generated using a standard zein protein by dilutions of stock solutions (1 mg/mL, each) dissolved in 65% ethanol or 95% ethanol, respectively. Specifically, 10 µL of standard zein protein dilution and zein protein extract were mixed with 200 µL of diluted protein assay dye reagent and incubated for 5 min at RT. The absorbance was measured at 595 nm using a Modulus microplate reader (Turner BioSystems, Sunnyvale, CA, USA).

### 2.3. SDS-PAGE Analysis

Sodium dodecyl sulfate-polyacrylamide gel electrophoresis (SDS–PAGE) was performed according to the Laemmli protocol [40] under reducing conditions for sample preparation. Prior to SDS gel, the extracted zein was precipitated by the addition of acetone in a 1:4 (*v/v*) excess and by incubation for 2 h at −20 °C. The mixture was centrifuged for 10 min at 15,000 rpm and left to dry overnight at RT. Prior to running the SDS-PAGE gel, the samples were dissolved in a sample buffer containing 2% (*w/v*) SDS, 10% (*w/v*) glycerol and 60 mM Tris, pH 6.8 and 0.01 % (*w/v*) bromophenol blue. The mixture was heated for 5 min at 55 °C. A total volume of approximately 25 μL each was loaded into the SDS gel. The gel was run for 15 min at 40 V and at 110 V for another 2 h. Proteins were stained overnight with Coomassie Brilliant Blue. A protein marker with molecular weights ranging from 10 to 200 kDa was used. The theoretical molecular weight from UniProt Knowledgebase was used to evaluate all SDS–PAGE data.

### 2.4. In-Gel Digestion of Zein Proteins and Zip-Tip Procedure

The gel bands were cut into small slices according to their molecular weight. The gel fragments were then washed in water for 15 min, and dehydrated in 3:2 ACN:MilliQ for 30 min. Following dehydration with ACN, the gel pieces were distained in 50 mM of NH_4_HCO_3_ (pH 8). The rehydration-dehydration process was done 2–3 times until the gel fragments were completely destained. Disulfide bridge reduction was achieved by the addition of 50 times molar excess of DTT followed by incubation at 56 °C for 1h. The samples were alkylated by incubation for 1h at RT in the dark with a 2.5 molar excess of IAA over DTT.

The gel fragments were lyophilized before being rehydrated on ice for 45 min using a cold solution containing 12.5 ng/L trypsin in 25 mM of NH_4_HCO_3_. After removing the excess enzyme solution, the gel sections were coated with 25 mM NH_4_HCO_3_ and incubated at 37 °C overnight. Proteolytic peptides were extracted by incubating gel pieces for 2h at RT with 3:2 ACN/0.1% TFA (*v/v*). The solution was collected and the process was performed twice.

Prior to MALDI-MS analysis, peptide and protein samples were desalted and concentrated using 10 μL pipette tips with ~0.6 μL C_4_ or C_18_ resin packed into the narrow end of the tip. ZipTipC_18_ and ZipTipC_4_ pipette tips were purchased from Millipore (ZipTip^®^ pipette tips). The wetting (50% ACN in MilliQ grade water) and equilibration (0.1% TFA) of the pipette tip resin, binding of peptides and/or proteins to the tip resin, removal of salts by washing, and lastly elution (0.1% TFA/50% ACN) of bound peptides were the five steps in the cleanup and desalting procedure.

### 2.5. Mass Spectrometric Analysis

The zein protein samples were analyzed by direct infusion ESI-Q-TOF using the Xevo G2 mass spectrometer (Waters, Milford, CT, MA). The data was acquired over m/z 50–1200 Da with a flow rate of 500 nl/min. A scan time of 0.5 s was used. The capillary voltage was 1.8 KV with the source temperature at 80 °C, the desolvation temperature at 15 °C with nanoglow gas pressure at 0.2 Bar, the desolvation gas flow at 600 L/Hr, the collision energy was set at 6.0 and the detector voltage was set at 3575. The instrument was calibrated with 1 pmol Glu-fibrinopeptide (Waters, Milford, CT, MA) prior to the samples being run, with an amino acid sequence EGVNDNEEGFFSAR and a double charged precursor ion of m/z 785.84.

MALDI-ToF MS analysis was performed on a Bruker Ultraflex MALDI ToF/ToF mass spectrometer operated in positive linear mode and equipped with a pulsed nitrogen UV laser (Bruker Daltonics, Hamburg, Germany). As matrix, a saturated solution of DHB dissolved in 3:2 ACN:0.1% TFA in MilliQ was used. The samples (0.7 µL) and matrix (0.7 µL) were spotted on a MALDI 384-spot target plate using the dried-droplet method. The mixture was allowed to co-crystallize and dry at RT.

### 2.6. Chemical Structure and Microtopography Analysis

Atomic force microscopy (AFM) surface images were obtained with a NTEGRA scanning probe microscope (NT-MDT Spectrum Instruments, Moscow, Russia). Rectangular silicon cantilevers NSG11 (NT-MDT, Russia) with two different tips of high aspect ratio (sharpened pyramidal tip, angle of nearly 20°, tip curvature radius of 10 nm and height of 10–15 µm) were used in order to minimize convolution effects. Here, we used the shorter (100 ± 5 µm) of those two. For sample preparation, 5 µL of the solutions were dropped onto the freshly cleaved mica surface and allowed to dry for 24 h at RT. All images were acquired in air, at room temperature (23 °C), in tapping mode, with the velocity of 6 mm/s. For image acquisition, the Nova v.19891 for Solver software was used.

Fourier Transformed Infrared Spectroscopy in the Attenuated Total Reflection (FTIR-ATR) measurements have been recorded at 4 cm^−1^ resolution with 140 scans recording, using an IR Tracer-100 FT-IR spectrometer (Shimadzu Corporation, Japan) equipped with a GladeATR module (PIKE Technologies, Madison, WI, USA). For each sample, the evaluations were made on the average spectrum obtained from three recordings. Background and sample spectra were obtained in the 400 to 4000 cm^−1^ wavenumber range.

## 3. Results

### 3.1. Proteomic Approach for Characterization of Zein Protein in Corn Flour Extraction

Zein, a complex protein, is one of the few grain proteins that can usually be isolated in a considerable amount from corn maize. Here, we investigated zein extraction from flours made by grinding maize kernel (KWS 3381), a trilinear, semi-latex, drought-resistant corn hybrid recommended for efficient grain yield. The process of extracting zein proteins from the hybrid strain began with grinding corn seeds, from which two fractions with 710 µm and 250 µm granulations size were selected for further studies. Both maize four, 710 µm and 250 µm granulations were suspended in two separate aqueous ethanol solutions, 65% and 95%, respectively, and the resulted samples were notated 65/250, 65/710, 95/250, and 95/710. Table 1 shows the efficacy of 15 min of extraction using either the ultrasonic bath or the ultrasonic homogenizer.

The ultrasonic homogenizer induced a faster and better extraction of zein as compared with the ultrasonic bath. The extraction time was previously optimized from 5 min to 30 min, and the better reproducibility was established for 15 min since we obtained different amounts of zein extracted between 5 and 10 min. As detailed in Table 1, ultrasonic homogenizer yielded a higher zein concentration than the ultrasonic bath, especially when flours with higher granulations were utilized (65/710 and 95/710).

The current methods for zein protein ultrasonic based extraction reported in the literatures [3,7,28,35] used an ultrasonic bath. As described by [35], the amount of zein protein extracted with 65% ethanol in 30 min using an ultrasonic bath and 250 µm maize granulation was just 0.27 mg/mL and 1.22 mg/mL for 710 µm maize granulation. By using an ultrasonic homogenizer, we considerably increase the amount of extracted zein proteins at 1.58 mg/mL and 2.09 mg/mL, respectively, in only 15 min sonication time.

Consequently, we further analyzed the protein content in the extracted fractions by using various analytical methods, such as SDS–PAGE electrophoresis, MS, AFM, and FTIR-ATR spectroscopy.

**Figure 1:**
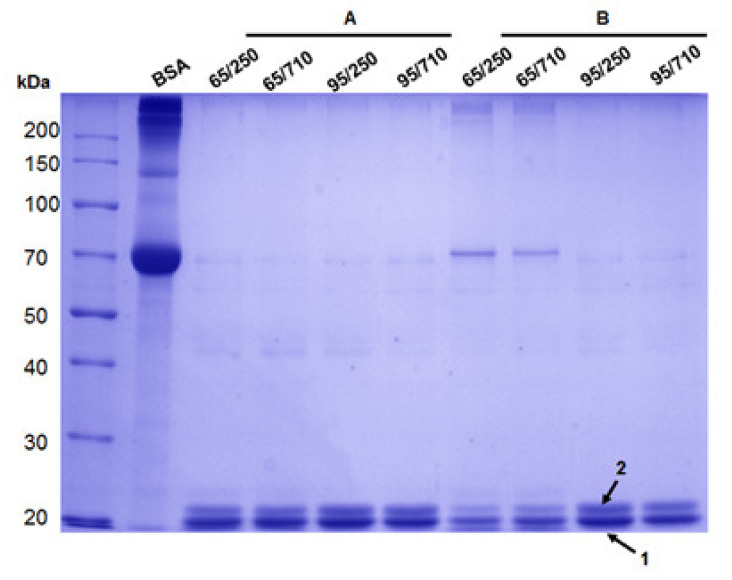
SDS-PAGE electrophoresis of extracted zein from maize inbred KWS 3381 after 15 min using ultrasonic bath (**A**) and ultrasonic homogenizer (**B**), where BSA: bovine serum albumin standard solution; 65/250: zein extracted with 65% ethanol solution from 250 µm flour granulation; 65/710: zein extracted with 65% ethanol solution from 710 µm flour granulation; 95/250: zein extracted with 95% ethanol solution from 250 µm flour granulation; and 95/710: zein extracted with 95% ethanol solution from 710 µm flour granulation.

The molecular weight of zein proteins was analyzed by SDS-PAGE, and despite the varying concentrations obtained after extraction, SDS gel was loaded with equal amounts (20 µg). As shown in Figure 1, two intense bands with different molecular weights, an MW of approximately 20 and 23 kDa, were observed, suggesting that the extracted zein was mostly α-zein monomer. The SDS profiles indicate comparable protein bands for all fractions, suggesting that in both 65% and 95% ethanol, similar zein protein content was extracted from maize flour with a granulation of 250 µm and 710 µm, respectively.

Additionally, in two fractions (65/250 and 65/710) using a lower aqueous ethanol solution of 65% ethanol, a visible protein band corresponding to approx. 70 kDa was obtained only for ultrasonic homogenizer extraction as a result of zein trimer formation. In all analyzed fractions, low abound protein bands were also observed at approx. 45 kDa due to the cysteine residues and their inter-chain disulfide-bridges formation.

For a precise identification of extracted zein, SDS-PAGE electrophoresis was followed-up by in gel digestion, MALDI-ToF, and electrospray ionization (ESI) mass spectrometry analysis.

**Figure 2:**
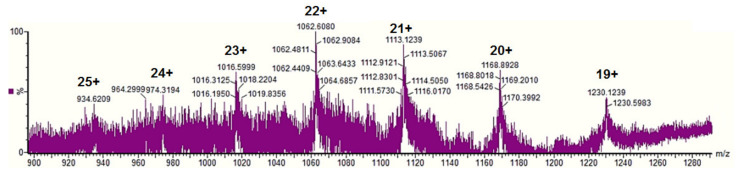
ESI mass spectrum of the extracted zein from the maize inbred KWS 3381 with 95 % ethanol in water from flour having 710 μm particle size.

Using ESI mass spectrometry, we analyzed the zeins extracted with 65% and 95% ethanol from the maize inbred KWS 3381 flour with the particles size 250 μm and 710 μm. As an example, the mass spectrum shown in Figure 2 corresponded to the zein extracted using an ultrasonic homogenizer with 95% ethanol from the 710 μm maize flour and displays peaks at m/z 974.31, 1016.59, 1062.60, 1113.12, 1168.89, and 1230.12 that were assigned as multiple charged ions of 19 kDa zein PMS2 (Uniprot accession no. *P24450*).

Additionally, the ESI mass spectrum of zein extracted using an ultrasonic bath with 95% ethanol from the 250 μm maize flour displayed peaks for two zein isoforms. The m/z at 1016.48, 1062.28, 1113.31, and 1459.29 were assigned to the multiple charged ions of 19 kDa zein PMS2 (Uniprot accession no. *P24450*) and m/z 1459.24, 1641.45, 1875.81, and 2188.43, corresponding to the multiple charged ions of Z1D alpha zein fragment (Uniprot accession no. *A8HNN3*) (data not shown).

Referring to the identified zein proteins, it must be notedthat the genomic data in the databases reflects the DNA sequence of a model plant, and that coding sequence changes exist between corn types. Consequently, the genome of the hybrid type (KWS 3381) studied in the present work hasnot yet been sequenced before, and our zein identification matched the zein existing sequences in the Uniprot protein database.

The identified zein proteins in ESI mass spectrum were partially confirm by MALDI-ToF MS after enzymatic digestion using trypsin. Even if zein proteins are not abundant in Lys and Arg residues, among the specific amino acids for trypsin cleavages, two resulted peptides were sufficient to match the zein primary amino acid sequence selected from Uniprot after ESI MS analysis.

**Figure 3:**
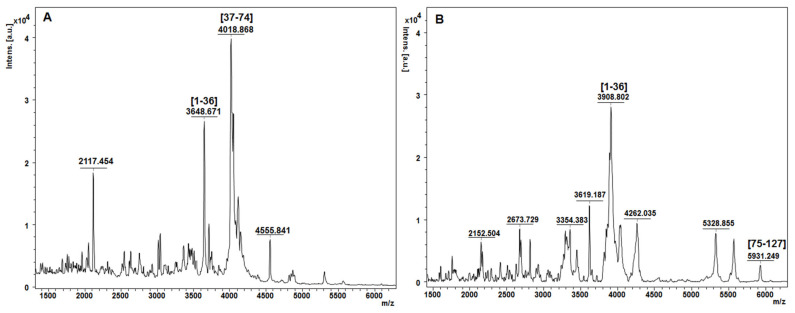
MALDI-ToF peptide mass fingerprint spectrum of zein proteins contained in band 1 (**A**) and band 2 (**B**) after in-gel digestion using trypsin. Labeled ion signals are peptide fragments corresponding to alpha-zein protein sequence.

In MALDI-ToF MS, a characteristic ion signal for bands 1 (Figure 1) was found at m/z 4018.51 and was assigned as 1–36zein protein fragment which corresponds to three zein isoforms (Uniprot accession no. *P24450, P02859* or *Q946V6*) with a theoretical calculated mass at m/z 4015.05 (Figure 3A).

In contrast, the MALDI ToF MS of the band 2 (Figure 1) indicated two tryptic peptide fragments [1–36] at m/z 3908.80 and [75–127] at m/z 5931.24, respectively, encoded by the *P04704* database entries (Figure 3B).

In this case, MS results effectively demonstrate the demand for complementary protein analysis using both ESI and MALDI ionization techniques, which gives interdependent information for protein identification at the amino acid level.

### 3.2. Chemical Structure and Microtopography Analysis

**Figure 4:**
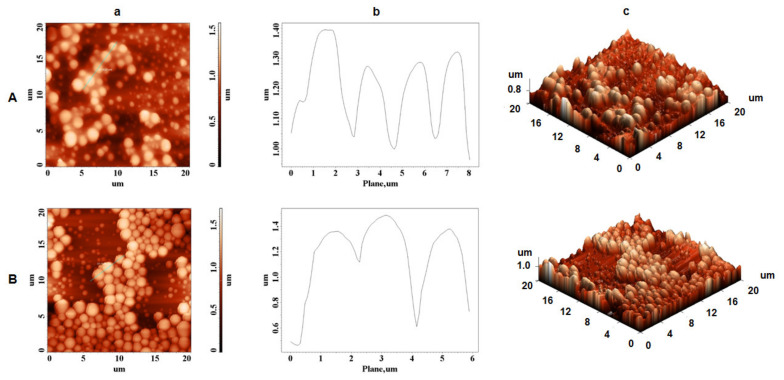
AFM images of extracted zein using ultrasonic homogenizer; (**A**) 65/250 (65% ethanol solution and 250 µm flour granulation) and (**B**) 65/710 (65% ethanol solution and 710 µm flour granulation); (**a**) height AFM images; (**b**) plot height distribution of particles along the profile line and (**c**) 3D AFM images.

AFM was developed and employed as a useful technique for detecting the surface topography of synthetic and biological materials at their molecular level. Therefore, in the current study, AFM analysis was used to investigate the microtopography of extracted zein and the potential self-assembly process using different ethanol concentrations and maize granulations. The two-dimensional height, plot height distribution of particles along the profile line, and three-dimensional (3D) AFM images were selected as parameters to illustrate the microtopography of extracted zein protein molecules. First, the fraction of 65% ethanol concentration and 250 µm maize granulation was analyzed by AFM, and the presence of densely packed spheres was observed as homogeneous layer (Figure 4A).

In contrast, maintaining the same ethanol concentration, but increasing the maize granulation at 710 µm, the zein particles were slightly changed in their surface topography (Figure 4B). As previously reported [41,42], the topographic modification of commercial zein (Sigma Aldrich) in stock solutions was attributed to various concentration of zein. As presented in Table 1, by using 65% aqueous ethanol solution and 710 µm maize granulation, a larger amount of zein was successfully extracted if comparing with the extraction of 250 µm maize granulation. Nevertheless, it is worth mentioning that the zein spheres at both 250 µm and 710 µm had a height of around 1.4 µm, about 1.3–1.9 µm, and 1.2–2 µm in width, respectively (Figure 4A-b and Figure 4B-b).

**Figure 5:**
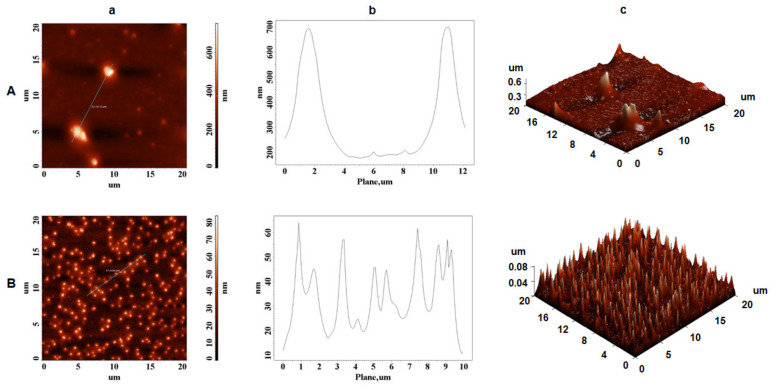
AFM images of extracted zein using ultrasonic homogenizer; (**A**) 95/250 (95% ethanol solution and 250 µm flour granulation) and (**B**) 95/710 (95% ethanol solution and 710 µm flour granulation); (**a**) height AFM images; (**b**) plot height distribution of particles along the profile line and (**c**) 3D AFM images.

Moreover, using 95% aqueous ethanol solution for zein extraction, surface topography differences were observed, as illustrated in Figure 5A,B, where irregular zeinshapes are present. The size of the zein globules were discontinuous, with a height of 0.7 µm (Figure 5A-b) and 0.06 µm (Figure 5B-b), and showed an average width of around 2.5 µm (Figure 5A-b) and 1 µm (Figure 5B-b). Those size differences may be influenced by the evaporation rate of the alcohol solution, since the structure of zein during liquid–liquid dispersion is a function of alcohol concentration [43]. Since different ethanol solutions evaporate faster than water solution, the polarity of the ethanol solution changed quickly as it evaporated, and high ethanol concentrations that present high hydrophobicity may not favor formation of β-sheets structure. Moreover, the morphology and structure of self-assembled α-zein previously revealed antiparallel β-sheets conformation in self-assembling zein nanospheres and nanofibers [44]. Kim et al. [45] found that the degree of aggregation of zein varied based on the binary solvent mixture’s composition, and as a result, the self-assembly mechanism of zein particles appears to be due not just to their amphiphilic properties, but also to the changing polarity of the solution as it evaporates.

Our studies confirm the experimental data reported by Zhong et al. [43], who suggested that at low zein mass fraction (0.2–5.0 mg/mL) in 70% ethanol, zein formed uniforms spheres, but as the zein concentration increased, spheres began to associate, melt, and deform, resulting in a variety of shapes.

However, in our work, zein forms different typology structures as the concentration of ethanol in the extraction solutions increases, which indicates a better homogeneity of the zein in a small percentage of ethanol (65%). Our data provides evidence that using 65% ethanol concentration and 710 µm granulation for zein extraction provides the optimal conditions for a homogeneous content that would allow these proteins to be used in future medicinal applications. This homogeneity must not be neglected when the simple α-zein molecule is designed to be a support for biomedical devices (antimicrobial films or drug delivery particles).

**Figure 6:**
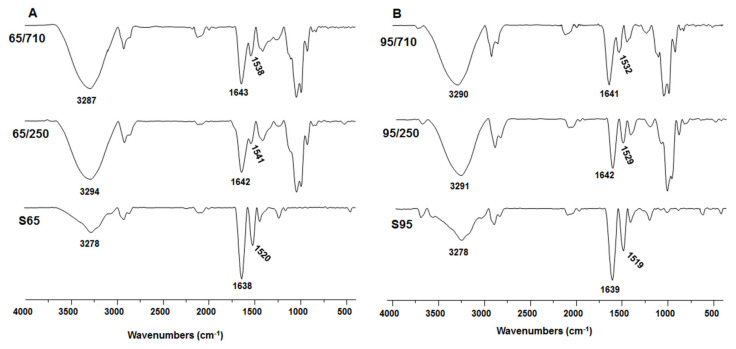
FTIR-ATR spectra of zein extracts by using ultrasonic homogenizer with (**A**) 65% ethanol aqueous and (**B**) 95% ethanol aqueous solution. S65: commercial zein in 65% ethanol; 65/250: extracted zein with 65% ethanol and 250 µm flour granulation; 65/710: extracted zein with 65% ethanol and 710 µm flour granulation; S95: commercial zein in 95% ethanol; 95/250: extracted zein with 95% ethanol and 250 µm flour granulation; 95/710: extracted zein with 95% ethanol and 710 µm flour granulation.

FTIR-ATR spectroscopy was used for the qualitative evaluation of the zein chemical modifications, using two different ethanol concentrations (65% and 95%) and two different maize flour granulations (250 µm and 710 µm) (Figure 6). All FTIR-ATR spectra were compared with commercial α-zein, noted S65 for 65% ethanol extraction and S95 for 95% ethanol extraction, respectively. The FTIR-ATR spectra are usually used to investigate the conformational changes of peptides and proteins, revealing their self-assembled abilities. The spectrum of extracted commercial zein (S65 and S95, as control) shows intense peaks characteristic of hydroxyl groups at 3278 cm^−1^. The presence of the amide I and amide II group can be proved by the peaks at 1638 cm^−1^ and 1520 cm^−1^ for S65 and 1639 cm^−1^, and 1520 cm^−1^ for S95.

The characteristic bands in FTIR-ATR spectra were also observed in the α-zein extract from hybrid (KWS 3381) corn with moderate shift. The hydroxyl stretching vibration peaks were observed to be hypochromic shifted after zein extraction using hybrid corn to 3294 cm^−1^ (Figure 6A-65/250) and 3287 cm^−1^ (Figure 6A-65/710) when using 65% ethanol, and to 3291 cm^−1^ (Figure 6B-95/250) and 3290 cm^−1^ (Figure 6B-95/710) when using 95% ethanol. Zein forms hydrogen bonds due to its amino, carboxyl, and amide groups. The formation of hydrogen bonds could possibly be followed by a self-assembly process, as shown by the shifting of the hydroxyl stretching vibration peak. Moreover, the amide I band is a significant and relatively intense band, mostly associated with the C=O stretching vibration and directly related to backbone conformation. The stretching vibration of C=O in peptide bonds (1600–1700 cm^−1^) appears to be related to its secondary structures, such as α-helix (1650–1660 cm^−1^), β-sheet (1610–1640 cm^−1^), and β-turn (1660–1700 cm^−1^), as well as inter- or intra-molecular aggregates (1640–1650 cm^−1^) of various protein fragments [46]. By comparing the spectra of the amide I and amide II signals, it was found that both were bathochromic shifted. These shifts could suggest that zein molecules have electrostatic contacts, showing that electrostatic interactions may play an important role in the self-assembly process.

## 4. Discussion

The novelty of these studies is the use of state-of-the-art protein separation and mass spectrometric protein identification methods to investigate the homogeneity of prolamins when extracted with either 65% ethanol or 95% ethanol in water, different granulations, and two homogenization protocols.

Our findings suggest that a combination of SDS-PAGE and high-resolution mass spectrometry is crucial for characterizing polymorphic protein composition in one dimensional gel band patterns by providing a better characterization of individual protein isoforms. The mass spectrometric resolution and peptide mass finger-print generated by trypsin digestion were adequate to identify one zein isoform from another that were closely separated in the SDS-PAGE gel and visualized by InstantBlue™ Coomassie Staining. Distinct α-zein proteins were identified in SDS-gel, corresponding to band 1 and band 2 for all experimental conditions used for protein extraction from hybrid (KWS 3381) maize, and additional oligomeric species were also observed mostly at lower ethanol concentration (65%) using harsh conditions (ultrasonic homogenizer based on microtip cell disrupter unit). The necessity for complementary protein MS analysis employing both ESI and MALDI ionization techniques, which provides interdependent information for protein primary amino acids sequence, was successfully demonstrated in this study when five α-zein isoforms were identified in database UniProtKB. Usually, the genomic information in the databases reflects the DNA sequence of a model plant, and that differences in coding sequences within varieties exist and should not be neglected when specific hybrid maize type are employed in the experimental work. The genomes of the hybrid varieties (KWS 3381) that have been investigated in this study have not yet been sequenced, and therefore, a comparison of experimental MS data matching the genomes is not currently possible.

Even though in the previously published research by Postu el al. [35], trypsin was replaced by chymotrypsin due to low Lys and Arg residues content in α-zein, in the present study, the “pure” generated peptide fragments by trypsin digestion were sufficient to identify the specific isoforms in KWS 3381 hybrid corn. The presence of a low content of basic residues in a protein is not suitable for an experimental proteomic approach using trypsin (trypsin cleaves at Lys and Arg C-terminus if the next amino acid in the peptide is not Pro), but is an important feature for forming drug-delivery particles resistant by digestive enzyme. Zein protein has generated a lot of attention in the industry [5] and biopharma [47], but we consider that the characterizations of employed zein proteins are critically required to control the shape and size of the final desired product. Since it is the most abundant protein (content ranges from 75 to 85%) in maize kernels [31], zeins extraction process was less complex, and eco-friendly by using human compatible organic solvent with no addition of reducing agents.

Moreover, AFM data have shown than zein forms different typologies structures as the concentration of ethanol in the extraction solutions increases, which indicates a better homogeneity of the zein in a small percentage of ethanol (65%). Our findings show that zein extraction with a 65% ethanol concentration and a 710 µm granulation yields the most uniform content, allowing these proteins to be employed in future medical applications. This homogeneity must not be neglected when the α-zein molecule is selected to be developed as a biocompatible support for biomedical devices (antimicrobial films or drug delivery particles). It is very important to control the films or spheres particle size when natural protein composition is applied as a major component of biomedical active compounds for human’s health and a specific characterization of the extracted protein is mandatory. Furthermore, FTIR-ATR studies have shown moderate bathochromic shifts of extracted α-zein, suggesting that α-zein molecules have electrostatic interactions which are required features for a spontaneous and natural self-assembly process.

Our results demonstrate the improvement of protein extraction leading to a homogeneous content of α-zein, the most abundant protein in corn flour. This new information allows us and other researchers to apply this eco-friendly extraction procedure for obtaining pure zein proteins that can be further used to broaden their applications for human health and environmental.

In summary, an optimized eco-friendly extraction approach for zein proteins from hybrid maize kernel was established here as a standard protocol for producing a homogeneous zein protein fraction. The homogeneity of extracted zein was confirmed using various analytical methods such as mass spectrometry, SDS-PAGE, AFM, and FTIR-ATR. All these methods comprised in a multidisciplinary study demonstrate a significant improvement in the characterization and identification of diverse endogenous zein proteins in maize.

## Figures and Tables

**Table 1:** Experimental characteristics and concentration (mg/mL) of zein protein extraction from maize kernels using 250 µm and 710 µm flour granulation, 65% and 95% ethanol concentration and ultrasonic conditions.

Extraction Method	Maize Sample	Flour Granulation (µm)	Ethanol Concentration (%)	Protein Concentration (mg/mL)
Ultrasonic homogenizer	65/250	250	65	1.58
	65/710	710	65	2.09
	95/250	250	95	1.44
	95/710	710	95	1.90
Ultrasonic bath	65/250	250	65	0.79
	65/710	710	65	0.63
	95/250	250	95	0.44
	95/710	710	95	0.28

## Data Availability

The authors confirm that the data supporting the findings of this study are available within the article and raw data are available from the corresponding author upon request.
